# Adhesion GPCRs are widely expressed throughout the subsections of the gastrointestinal tract

**DOI:** 10.1186/1471-230X-12-134

**Published:** 2012-09-25

**Authors:** Luca Badiali, Jonathan Cedernaes, Pawel K Olszewski, Olof Nylander, Anna V Vergoni, Helgi B Schiöth

**Affiliations:** 1 Department of Neuroscience, Uppsala University, BMC, Uppsala, SE 75124, Sweden; 2 Department of Biomedical Sciences, University of Modena and Reggio Emilia, Via Campi, 41125, Modena, Italy; 3 Department of Biological Sciences, University of Waikato, Hamilton, New Zealand

## Abstract

**Background:**

G protein-coupled receptors (GPCRs) represent one of the largest families of transmembrane receptors and the most common drug target. The *Adhesion* subfamily is the second largest one of GPCRs and its several members are known to mediate neural development and immune system functioning through cell-cell and cell-matrix interactions. The distribution of these receptors has not been characterized in detail in the gastrointestinal (GI) tract. Here we present the first comprehensive anatomical profiling of mRNA expression of all 30 *Adhesion* GPCRs in the rat GI tract divided into twelve subsegments.

**Methods:**

Using RT-qPCR, we studied the expression of *Adhesion* GPCRs in the esophagus, the corpus and antrum of the stomach, the proximal and distal parts of the duodenum, ileum, jejunum and colon, and the cecum.

**Results:**

We found that twenty-one *Adhesion* GPCRs (70%) had a widespread (expressed in five or more segments) or ubiquitous (expressed in eleven or more segments) distribution, seven (23%) were restricted to a few segments of the GI tract and two were not expressed in any segment. Most notably, almost all Group III members were ubiquitously expressed, while the restricted expression was characteristic for the majority of group VII members, hinting at more specific/localized roles for some of these receptors.

**Conclusions:**

Overall, the distribution of *Adhesion* GPCRs points to their important role in GI tract functioning and defines them as a potentially crucial target for pharmacological interventions.

## Background

The superfamily of G protein-coupled receptors (GPCRs) is one of the largest families of membrane bound proteins in the human genome [1] comprising about 800 members. Being involved in a high number of physiological functions, including development, neurotransmission, metabolism, reproduction, immune responses, and behavior, GPCRs act as receptors for a great number of different signals, both endogenous - amines, peptides, proteins, lipids, nucleotides, neurotransmitters - and sensory, as organic odorants, pheromones, tastes and photons. According to the phylogenetic analysis of the entire human GPCR repertoire, five subfamilies make up the GRAFS classification system: *Glutamate*, *Rhodopsin*, *Adhesion*, *Frizzled/Taste2* and *Secretin*[2]. The phylogenetic grouping of *Adhesion* GPCRs, based on the 7TM regions, revealed that there are seven groups [3].

GPCRs are characterized by having seven α-helices that span the plasma membrane and form a receptor with a binding cavity for a ligand; the extracellular segment may also be able to bind a ligand. The main feature of the *Adhesion* family is the long N terminus with complex domain architecture which is thought to be highly glycosylated and form a rigid structure in the outer part of the protein. This extracellular portion contains the GPCR proteolytic site (GPS) and several various domains that can also be found in other proteins such as lectin, epidermal growth factor, olfactomedin, immunoglobulin, thrombospondin and cadherin domains [3]. The GPS domain is referred to as an intracellular cleavage motif, pivotal for the protein transport from the endoplasmic reticulum to the membrane [4], while several other N terminal domains play important roles in the receptor-ligand binding as well as cell-to-cell and cell-to-matrix adhesion [5]. Distinguishing themselves even more from other GPCRs, *Adhesion* GPCRs are genomically complex, each receptor having many isoforms [6], with multiple alternatively-spliceable introns and large genomic sizes, which makes them difficult to study [7]. Only a few members of *Adhesion* GPCRs have been demonstrated to interact with G proteins [8,9].

Beyond its evident role in digestion and adsorption, the gastrointestinal (GI) tract is involved in a variety of other physiological functions, such as endo- and exocrine secretion and immune responses [10-12]. Its autonomous neuronal network, referred to as the enteric nervous system or ENS, is also intimately linked with the brain in the brain-gut axis important for, among others, food intake regulation [13,14]. GPCRs in the GI tract are known to be involved in nutrient balancing [15-17] and regulation of the immune system [18,19]. In some cases their gross expression patterns in the GI tract have been established, but more subtle proximodistal variations in expression and their biological functions have yet to be determined [20]. Furthermore, dysfunction of GPCRs is already known to contribute to certain diseases affecting the GI tract. For example, *GPR49* overexpression has been related to increased incidence of human colon primary tumors [21], whereas overexpression of the *Adhesion* GPCR *CD97* has been correlated to colorectal cancer [22], rectal adenocarcinoma recurrence and metastasis [23], and gastric carcinoma [24].

Though about 85% of *Adhesion* GPCRs are still orphans or lack biological characterization (details about known functions are provided in [6]), EMR receptors have been shown to be involved in immune responses [25]: Crohn’s disease and ulcerative colitis are two of the main inflammatory bowel diseases affecting the GI tract, and there is a growing body of evidence indicating that the onset of these pathologies may be related to an immune system deregulation by the ENS or gut microbiota [11,12]. The characterization of immunologically important *Adhesion* GPCRs in the GI tract may therefore lead to a greater understanding of these pathological conditions. Profiling of the entire GI tract, accounting for intra-regional differences is important since proximal-distal part of GI tract’s anatomical regions can serve different roles or be selectively affected by diseases; e.g. bile acids and vitamin B12 adsorption occur in the distal ileum, Barrett’s disease affects the distal esophagus [26], and gastric cancer occurs either proximally or distally [27]. Likewise, Crohn’s disease more frequently affects the terminal ileum [28]. Knowledge of the proximodistal expression pattern may therefore indirectly facilitate pinpointing the function of *Adhesion* GPCR orphans.

We present here the first complete analysis of mRNA expression of all members of the *Adhesion* GPCRs subfamily throughout the entire rat GI tract, which was divided into twelve subsegments (as described previously [29]). Using RT-qPCR with a validated range of housekeeping genes, we studied the expression in the esophagus, the corpus and the antrum of the stomach, the proximal and distal parts of the duodenum, jejunum, ileum and colon, and in the cecum.

## Methods

### Animal handling and tissue isolation

**Figure 1:**
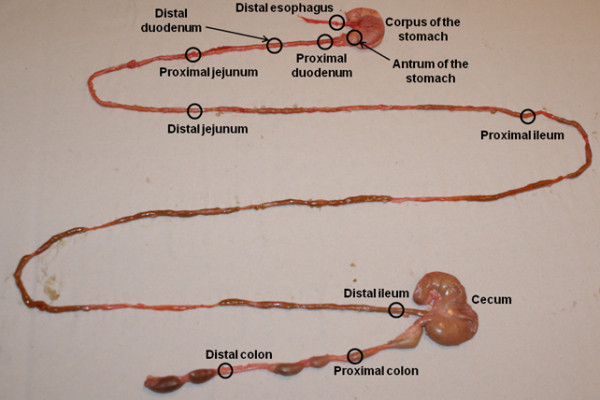
**GI sectioning approximately indicating the different sections of the rat GI tract used for analysis.** The GI tract was divided into twelve segments: the esophagus, the corpus and the antrum of the stomach, the proximal and distal parts of the duodenum, ileum, jejunum and colon, and the cecum.

Three male Dark Agouti rats (Scanbur AB, Sweden) weighing approximately 200 grams, were kept under constant conditions (12 h dark/light cycle) at 21°C. The animals were fasted overnight but had free access to water before carrying out the experiment. The following morning the animals were intraperitoneally anesthetized with Na-5-ethyl-1-(1’-methyl-propyl)-2-thiobarbituric acid (Inactin®; 125 mg/kg b. wt). Body temperature was maintained at 37.5 ± 0.5°C through a temperature regulator controlling a heating pad. Thereafter, a tracheotomy was performed and a cannula (PE-200) was inserted to guarantee free airways. Following a midline incision to open the abdominal cavity, the following structures, about 5 to 10 mm in length, were localized and dissected (Figure 1): Distal esophagus (a few mm from the stomach); corpus of the stomach; antrum of the stomach; proximal (1 mm from the pylorus) and distal duodenum (4 cm from the pylorus); proximal (9 cm from the pylorus) and distal jejunum (19 cm from the pylorus); proximal (29 cm from pylorus) and distal ileum (2.5 cm from the ileocecal valve); cecum; proximal (5 cm from ileocecal valve) and distal colon (12 cm from the ileocecal valve). The whole GI tract wall was isolated for the RT-qPCR analysis.

After the operation, an intravenous bolus injection of a saturated KCl solution was used to euthanize the animals. All animal procedures followed the regulations and policies outlined by the Swedish Animal Protection Act and were approved by the Uppsala Ethic Committee.

### RNA isolation and cDNA synthesis

RNA isolation and cDNA synthesis were performed as previously reported [30]. Briefly, tissue samples were homogenized by sonication in the TRIzol reagent (Invitrogen, Sweden) using a Branson sonifier. Chloroform was added to the homogenate and centrifuged at 12000 g at 4°C for 15 minutes. The aqueous phase was collected and RNA precipitated with isopropanol. The pellets were washed with 75% ethanol, air dried and dissolved in RNAse-free water. The DNAse treatment was performed to remove DNA contamination: DNAse I (Roche Diagnostics, Sweden) was added to the samples and incubated at 37°C for 4 h, followed by inactivation by heating to 75°C for 15 minutes. Absence of DNA contamination in the RNA samples was confirmed by PCR. Nanodrop® ND-1000 Spectophotometer (NanoDrop Technologies, Delaware, USA) was used to determine RNA concentration. Thereafter, cDNA was synthesized by priming with random hexameres and MMLV reverse transcriptase (GE Healthcare, Sweden). PCR was performed to confirm cDNA synthesis. An equal amount of the total cDNA from each of the three rats was pooled, and the pooled solution was used as the template material for RT-qPCR.

### Primer design

Sequences for rat housekeeping genes and all known *Adhesion* GPCR gene sequences were downloaded from the GenBank. All primers were designed using Beacon Primer Design 7.0 (Premier Biosoft, USA) and positioned within TM regions of the *Adhesion* GPCRs. The primer sequences for rat *Adhesion* GPCRs and housekeeping genes are provided in additional material (see Additional file 1).

### Quantitative real-time PCR

The RT-qPCR was performed using a MyiQ thermal cycler (Bio-Rad Laboratories, Sweden). Each RT-qPCR reaction, with a total volume of 20 μl, contained cDNA synthesized from 25 ng of total RNA, 0.25pmol/μl of each primer, 20 mM Tris–HCl (pH 8.4), 50 mM KCl, 4 mM MgCl2, 0.2 mM NTP, SYBR Green (1:50000) (Invitrogen, USA) and 0.02U/μl Taq DNA polymerase (Biotools, Spain). The reaction conditions were the following: initial denaturation at 95°C for 4 min, succeeded by 40 cycles at 95°C for 15 s, 55–62°C for 30 s (optimal annealing temperature) and 72°C for 30 s. This was followed by 81 cycles at 55°C for 10 s (increased by 0.5°C per cycle). All real-time PCR experiments were run in triplicates. A negative control for each primer pair and a positive control with 25 ng of rat genomic DNA was included on each plate.

### Data analysis and relative expression calculations

Bio-Rad iQ5 software v2.0 software (Bio-Rad Laboratories, Sweden) was used to process RT-qPCR data and obtain threshold cycle (Ct) values. Melting curves were analyzed to assure that only one product with the expected melting point was amplified and that this was separate from the negative control. LinRegPCR was used to calculate PCR efficiencies for each sample and Grubbs' test (GraphPad, USA) was applied to exclude any outliers when calculating the average PCR efficiency for each primer pair. The delta Ct method [31] was used to convert Ct values into relative quantities with the standard deviation, and the highest expression was normalized to 1. The GeNorm software [32] was used with results from the five most stable housekeeping genes to calculate normalization factors for each tissue to compensate for differences in cDNA quantity. Subsequently, the normalized quantities were calculated and maximum expression was set to 1: all relative expression values are shown as fold decrease with respect to the detected maximum expression (see Additional file 2).

## Results

The rat *Adhesion* GPCR gene sequences were downloaded from GenBank (see Additional file 1) and 7TM regions were identified with the Conserved Domain Database [33]. We analyzed the expression of 30 *Adhesion* GPCR family members in the rat GI tract. The gut was divided into twelve different segments proceeding from the esophagus to the colon and each tissue was isolated and used for RNA extraction and cDNA synthesis. The expression values of five housekeeping genes (*Histone protein 3b*, *β-tubulin*, *β-actin*, *succinate dehydrogenase complex*, *subunit A* and *cyclophilin*) were used to calculate normalization factors for rat cDNA. Each reaction was run in triplicate and with a positive control (genomic DNA) to confirm the validity of the amplification process; relative expression values for rat *Adhesion* GPCRs are displayed as a fold decrease relative to the detected maximum expression, arbitrarily set at 1 (see Additional file 2).

**Figure 2:**
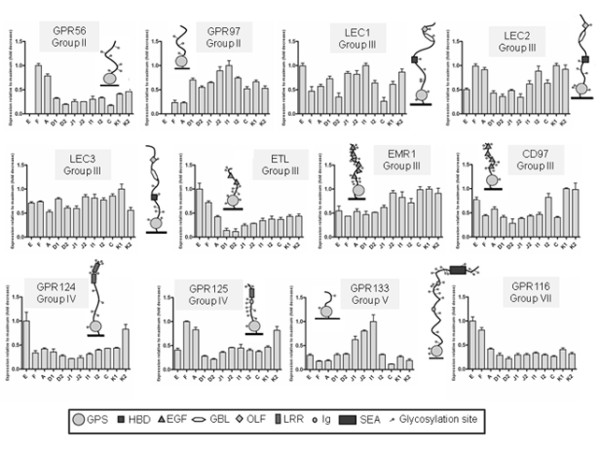
**GPCRs expressed ubiquitously along the GI tract.** Each panel refers to one GPCR showing three pieces of information: group of belonging, N terminal moieties and relative expression in twelve segments of the GI tract. Expression levels are relative for each gene (maximal level of expression set to 1). Values are plotted as the mean ± SD; n = 3. The phylogenetic grouping is based on the 7TM regions; the schematic representation of the domains in the N-termini as determined by RT-BLAST at NCBI is adapted from our previous work [3]. Abbreviations for N-termini moieties: GPS, GPCR proteolytic site; HBD, hormone-binding domain; EGF, epidermal growth factor; OLF, olfactomedin; GBL, galactose-binding lectin domain; LRR, leucine rich repeats; Ig, immunoglobulin; SEA, sperm protein, enterokinase, and agrin; glycosylation sites (NXS or NXT tripeptide sequences that conform to the consensus sequence for N-linked glycosylation) – shown as small circles attached to the N-termini stretches; E, esophagus; F, corpus of the stomach; A, antrum of the stomach; D1 and D2, proximal and distal parts of the duodenum; J1 and J2, proximal and distal parts of the jejunum; I1 and I2, proximal and distal parts of the ileum; C, cecum; K1 and K2, proximal and distal parts of the colon.

**Figure 3:**
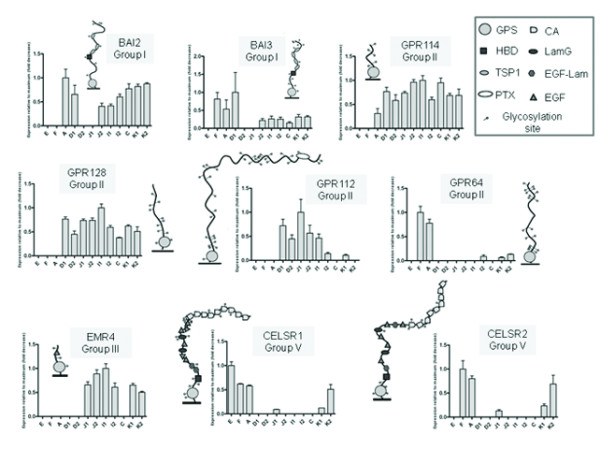
**GPCRs with widespread expression along the GI tract.** Each panel refers to one GPCR showing three pieces of information: group of belonging, N terminal moieties and relative expression in twelve segments of the GI tract. Expression levels are relative for each gene (maximal level of expression set to 1). Values are plotted as the mean ± SD; n = 3. Abbreviations for N-termini moieties: GPS, GPCR proteolytic site; HBD, hormone-binding domain; TSP1, thrombospondin; PTX, pentraxin domain; EGF, epidermal growth factor; CA, cadherin domains; LamG, laminin; glycosylation sites (NXS or NXT tripeptide sequences that conform to the consensus sequence for N-linked glycosylation) – shown as small circles attached to the N-termini stretches; E, esophagus; F, corpus of the stomach; A, antrum of the stomach; D1 and D2, proximal and distal parts of the duodenum; J1 and J2, proximal and distal parts of the jejunum; I1 and I2, proximal and distal parts of the ileum; C, cecum; K1 and K2, proximal and distal parts of the colon.

**Figure 4:**
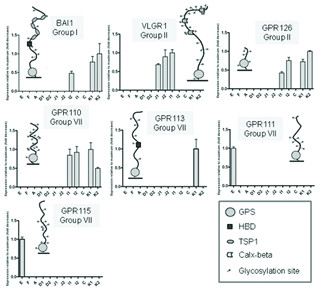
**GPCRs with limited expression along the GI tract.** Each panel refers to one GPCR showing three pieces of information: group of belonging, N terminal region and relative expression in twelve segments of the GI tract. Expression levels are relative for each gene (maximal level of expression set to 1). Values are plotted as the mean ± SD; n = 3. *GPR111*, *GPR113*, *GPR115* were only expressed in one sub-segment: the expression value was arbitrarily set to 1 to indicate the presence of expression. Abbreviations for N-termini moieties: GPS, GPCR proteolytic site; HBD, hormone-binding domain; TSP1, thrombospondin; PTX, pentraxin domain. Glycosylation sites (NXS or NXT tripeptide sequences that conform to the consensus sequence for N-linked glycosylation) are shown as small circles attached to the N-termini stretches; E, esophagus; F, corpus of the stomach; A, antrum of the stomach; D1 and D2, proximal and distal parts of the duodenum; J1 and J2, proximal and distal parts of the jejunum; I1 and I2, proximal and distal parts of the ileum; C, cecum; K1 and K2, proximal and distal parts of the colon.

A total of twelve GPCRs (40%) were found to be ubiquitous along the GI system (expressed in at least eleven segments – Figure 2), nine GPCRs (30%) had widespread expression (detected in at least five segments – Figure 3), seven GPCRs (23%) had restricted expression (transcript found in no more than four segments – Figure 4) and two GPCRs were not detected in any segments. The ubiquitously expressed genes (Figure 2) consisted of two members from Group II, namely *GPR56* and *GPR97* (although neither was expressed in the esophagus), all but one (*EMR4*) of the members from Group III (*LEC1*, *LEC2*, *LEC3*, *ETL*, *EMR1*, *CD97*), two members from Group IV (*GPR124* and *GPR125*; the third member *GPR123* could not be detected in any segment), *GPR133* from Group V, and *GPR116* from Group VII.

Among genes with widespread expression (Figure 3), *BAI2* and *BAI3* displayed a very similar pattern, being detected in the antrum of the stomach and the proximal part of the duodenum, and from the distal jejunum to the colon. In comparison, *BAI1* had limited expression, only being detectable in the distal ileum and colon. *GPR114* was found throughout the entire GI tract except for the esophagus and fundus of the stomach, while *GPR128* was expressed in the whole intestine, but not in the stomach or esophagus. *GPR112* was found in the duodenum, jejunum, ileum and proximal colon. *GPR64* was expressed only in the stomach and, to a lesser extent, in the distal ileum and colon. *CELSR1* and *CELSR2*, members of Group V, displayed a very similar pattern, being expressed in the stomach, colon and proximal jejunum, while *CELSR1* was also found in the esophagus. No expression of *CELSR3* could be detected. *EMR4* expression was restricted to the jejunum, ileum and colon. Out of the nine widespread *Adhesion* GPCRs, as many as four belong to Group II.

In total, seven genes had restricted expression (Figure 4), including the aforementioned BAI1. Two genes were members of Group II: *VLGR1*, with increasing expression from the jejunum to the proximal part of ileum, and *GPR126*, with isolated expression in the ileum and colon. An expression pattern similar to that of the latter gene was also observed for *GPR110*, belonging to Group VII, while from the same group, *GPR111* and *GPR115* were only expressed in the esophagus, and *GPR113* expression was detected at a low level only in the proximal colon. Out of the seven genes with restricted expression, the majority (4 out of 7) thus belonged to Group VII.

## Discussion

In this paper, we present the first comprehensive chart of the mRNA expression of all 30 *Adhesion* GPCRs in the rat GI tract. We find that the majority of the GPCRs are expressed ubiquitously throughout the GI tract, highlighting their importance for GI tract physiology. At the same time, the restricted expression patterns of seven GPCRs suggest that these receptors may have specific functions in these parts of the GI tract and they in particular may constitute potential therapeutic targets. While two other studies have mapped GPCRs expression in the mouse gut [34,35] as well as one study that mapped the *Adhesion* GPCRs expression distribution in the mouse and rat [36], in none of these studies has the GI system been divided into so many segments, using both proximal and distal subsegments of several GI tract regions. Based on our results we found that the *Adhesion* GPCRs could be divided into three categories: genes ubiquitously expressed, genes with widespread expression, and genes with restricted/specific expression.

### Genes with ubiquitous expression

Twelve GPCRs (namely *GPR56*, *GPR97*, *LEC1*, *LEC2*, *LEC3*, *ETL*, *EMR1*, *CD97*, *GPR124*, *GPR125*, *GPR133* and *GPR116*) were detected in at least eleven segments along the GI tract (Figure 2). Being ubiquitously expressed, these genes may play important roles for normal GI tract functioning. Intriguingly though, most of these receptors still have no known functions. Among the few de-orphanized GPCRs, *GPR56* is known to be involved in the development of the testis [37], and CNS [38,39]. Similarly, *GPR56* might be involved in the development of the GI tract. Furthermore, overexpression of *GPR56* has been reported in esophageal squamous cells carcinomas (EECCs) and dysplastic tissues contrary to adjacent nonmalignant esophageal tissue [40]. As we found no esophageal expression in wild type animals, but high gastric (especially fundus) expression, our results lends further support to the conclusion by Sud et al. that *GPR56* is an interesting candidate as an early diagnostic marker in esophageal cancer.

CD55 and chondroitin sulphate have been described as ligands for CD97 leading to leukocyte activation [41]: lack of CD55 and CD97 resulted in decreased arthritis in mouse experimental models of rheumatoid arthritis [42]. *CD97* has also been implicated in the initiation and establishment of inflammatory processes, e.g. in multiple sclerosis [43]. Outside cells of the immune system, *CD97* is also expressed in smooth muscle cells [44,45]. Since we detected the transcript throughout the whole GI tract, probably also in associated immune cells, we suggest *CD97* might constitute a target in inflammatory bowel diseases, in particular Crohn’s disease that is able to affect any region of the GI tract [46]. Interestingly, all but one of the members of the group III members were ubiquitously expressed, which could imply that this group of Adhesion GPCRs is of particular importance for GI tract functioning.

### Genes with widespread expression

Nine genes (*BAI2*, *BAI3*, *GPR114*, *GPR128*, *GPR112*, *GPR64*, *EMR4*, *CELSR1* and *CELSR2*) were found to have widespread expression, that is, they were detected in more than five segments (Figure 3). *BAI2* and *BAI3*, belonging to the family of brain angiogenesis inhibitor (BAI), displayed very similar expression patterns. Therefore, they might serve similar roles, such as being involved in angiogenesis in the GI tract, which is pivotal in ischaemia and tumorigenesis, and which has recently been shown to be of importance for the development and perpetuation of IBD [47,48]. *GPR64* is known to interfere with fluid re-adsorption [49] and our results showed high expression of the *GPR64* transcript in the stomach but not in adjacent segments. We therefore speculate that *GPR64* might be important for water homeostasis during the first digestive process in the stomach where water, together with mucus, HCl and pepsinogen, are secreted.

From the *CELSR family, CELSR2* and *CELSR3* are known to be distributed mainly in CNS and to regulate neural development [50]. In contrast, *CELSR1* has been described in lungs, involved in spatial development and branching morphogenesis [51]. Given that *CELSR1* and *CELSR2* had such limited but very similar expression in the GI tract, we hypothesize that these two genes might control the reciprocal signaling interactions between the epithelium and mesenchyme during morphogenesis of gastric epithelia and their expression is maintained also in the adult stage. *GPR112* has been reported in the dominant neuroendocrine cells of the GI tract, the enterochromaffin (EC) cells, in the normal mucosa of the human ileum, and also as a potential target for GI neuroendocrine carcinomas [52]. We detected its transcript in the duodenum - where EC cells display the highest expression [53] - jejunum, ileum and proximal colon: our finding is in good agreement with the work of Ito et al. [35]. *GPR112* could be involved in serotonin release or participate in the nutrient sensing functions of EC cells [54].

### Genes with restricted expression

Seven *GPCRs* (*BAI1*, *VLGR1*, *GPR126*, *GPR110*, *GPR113*, *GPR111* and *GPR115*) were found to be expressed in fewer than five segments (Figure 4), each showing highly specific and varying expression patterns. *BAI1* is known to be involved in angiogenesis, tumor formation [55] and host responses to Gram-negative infections [56], which include severe ICU-acquired infections [57,58]. Given the restricted expression in the colon and ileum, this receptor may play a role in tumor development in the intestine and in defense mechanisms against Gram-negative bacteria in the lower GI tract. *GPR126* has been reported to play an essential role for peripheral nerve development and myelination in mammals [59]; its limited expression in the ileum and colon could suggest an involvement in myelination of neurons of the myenteric plexuses crucial for GI motility, or submucosal plexuses, which regulate luminal and epithelial cell function. Mutations on *VLGR1* are known to underlie human Usher syndrome type II [60]. The complex extracellular domain of the *VLGR1* receptor, with a Calx-β cation binding motif, has led to a suggested role in the sensing of Ca^2+^[61]. Given that *VLGR1* expression was restricted to the jejunum and proximal ileum, one might hypothesize that *VLGR1* participates in Ca^2+^-sensing in the small intestine. *GPR110* has been recently shown to be an oncogene in murine T lymphomas and a marker for lung and prostate cancer [62]. The receptor is orphan, and since the expression was restricted to the ileum and colon, *GPR110* could potentially play a role in sensing nutrients or in malignancies, such as Coeliac disease-associated T lymphomas [63] or cancer of the small or large intestine.

**Figure 5:**
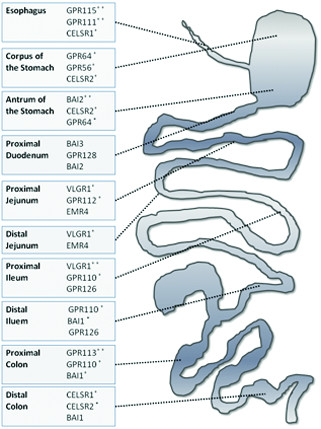
**GPCRs whose high expression is associated with specific subsegments of the GI tract.** The figure shows GPCRs whose levels were found to be markedly higher in a given segment relative to the expression level in other adjacent segments. For each region, two or three genes with the highest regionally specific expression levels are shown. To better illustrate the extent to which a given gene is highly expressed in a subsegment-specific manner compared with adjacent segments, a three-level system was adopted using the * symbol, assigning none, one or two symbols to each gene. The greater the deviation in expression level is from the surrounding expression pattern, the more asterisks have been assigned for a given gene. It should be noted that the distal duodenum and the cecum are not shown in the figure since no region-specific high gene expression levels were found for these two segments.

There were consistent differences in the expression between the proximal and distal part of the same segment for a given gene, as shown in Figure 5, which highlights GPCRs highly expressed in specific subsegments of the GI tract. For example, *VLGR1* had its highest expression in the proximal ileum, but it was not detected in the distal segment (Figure 4). This observation is in accordance with our previous work [29], describing notable proximodistal differences in solute carrier profiling in the GI tract. Our results therefore further support the rationale of analyzing each segment not as a homogenous entity but taking into account proximodistal differences. It is worth noting that members of the same group, e.g. Group II members *GPR114* and *GPR64* or Group VII members *GPR111* and *GPR113*, are expressed in different segments, thus they might regulate different functions.

It should be also noted that the direct comparison between our findings and previous studies may not always be possible, mainly due to the species used (mouse vs rat) and dissection differences, as other authors [35] analyzed the mucosa and muscle separately without taking into account the proximodistal subsegmentation of the GI tract. Our results are however consistent with the result previously obtained by our group [36]: genes that had been identified as being expressed along the GI tract had their expression pattern confirmed, though in greater detail. Examples of these genes include GPR133, GPR124, GPR125 and all members of Group III. In line with the earlier report, we did not detect *GPR112*, *GPR126* or *GPR128* in the stomach. Even so, there are some discrepancies, for example *CELSR2* (in contrast to *CELSR1*) was not detected by Haitina and coworkers, whereas herein we detected both *CELSR* receptors, identifying a very similar pattern (Figure 3). This can be explained by considering that a relatively low gene expression in the GI tract relative to other tissue types could have prompted a conclusion that the expression level in the gut is negligible. Compared to the findings by Ito et al., we found no expression of either *BAI1* or *GPR126* in the upper intestine – this may be due to the different species used [35]. It should be emphasized, however, that for many other GPCRs such as *BAI2*, *BAI3*, *CD97*, *GPR111* and *GPR112*, our results confirm and refine the expression patterns found by the aforementioned group. The results might have been somewhat affected by the timing of the experiments or other conditions, such as an overnight fast. Furthermore, mRNA expression does not always correspond to protein expression [64,65]. Finally, GPCRs are known to undergo extensive alternative splicing [3] that generates multiple transcripts including soluble forms: primers used in this study are located in the 7TM regions and thus might not cover the entire transcript population.

## Conclusions

Taken together these results indicate that, compared to solute carriers, *Adhesion* GPCRs display more restricted expression patterns in the GI tract [29], suggesting that *Adhesion* GPCRs play a more specific role in the GI tract, a role that could be important both for physiological or pathological conditions. This notion is further strengthened by the fact that *Adhesion* GPCRs are known to be restricted to certain cell types and to be highly regulated in their expression, likely serving distinct physiological functions [6]. We should therefore consider that GPCRs represent an unexploited potential for drug targeting [66]. However, given that GPCRs constitute the most common drug target (36%), but less than 82 distinct GPCRs are targeted, this clearly highlights the importance of exploring *Adhesion* GPCRs as potential drug targets.

In summary, our study shows that about 70% of the rat *Adhesion* GPCRs display a widespread or ubiquitous distribution while about a quarter of the studied receptors had limited expression in the GI tract. Their extensive distribution suggests a fundamental role of this receptor family.

## Competing interests

The authors declare that they have no competing interests.

## Authors’ contributions

LB carried out the RT-qPCR study, performed the statistical analysis and drafted the manuscript. JC participated in the design of the study and help to draft the manuscript. PKO participated in the design of the study and help to draft the manuscript. ON carried out histological dissections and tissue preparation. AVV participated in the design and coordination of the study. HBS conceived the study, and participated in its design and coordination and helped to draft the manuscript. All authors read and approved the final manuscript.

## Pre-publication history

The pre-publication history for this paper can be accessed here:

http://www.biomedcentral.com/1471-230X/12/134/prepub

## Supplementary Material

Additional file 1 **Primers used for real-time PCR analysis.** The table includes gene names for rat (r) *Adhesion* GPCRs and house-keeping genes (*), GenBank accession numbers, primer sequences and the expected size of the PCR products . NA – not available. Click here for file

Additional file 2 **Summary of relative expression of all *Adhesion *GPCR members.** Expression levels relative for each gene (maximal level of expression set to 1; n.d., not detected after 40 cycles of the PCR). The phylogenetic grouping is based on the 7TM regions [3]. *GPR111*, *GPR113*, *GPR115* were only expressed in one sub-segment: the expression value was arbitrarily set to 1 to indicate the presence of expression. *GPR133* and *CELSR3* could not be detected in any segment. Click here for file
