# Supplementary material for: Adhesion GPCRs are widely expressed throughout the subsections of the gastrointestinal tract
Source: BMC Gastroenterol. 2012 Sep 25;12:134. doi: 10.1186/1471-230X-12-134 (PMC3526421; doi:10.1186/1471-230X-12-134)
Supplement: Additional file 1 — Primers used for real-time PCR analysis. The table includes gene names for rat (r) Adhesion GPCRs and house-keeping genes (*), GenBank accession numbers, primer sequences and the expected size of the PCR products . NA – not available. [file 1471-230X-12-134-S1.pdf]

Name	Accesion No.	5’	3’	Size (bp)
rBAI1	XM_343260	tggatgtctgctgtgcttgctg	acgatgacgaagccctccagtg	95
rBAI2	XM_232778	tcctgcgtggtactgcctctcc	gcacggcggtgatgacaaagc	136
rBAI3	XM_217367	gacagataaacgctccatattg	acctctctccgaagaatgc	102
rCD97	NM_001012164.1	ctgcctgctgctctgtatcc	cccttcattctcaacaccaacc	136
rCELSR1	XM_001078424	tggtgtccttcgtcctgctctc	agccgcaatgaggttcttgtgg	80
rCELSR2	XM_001070611	gcacttcctgtacctttg	agcatgtagtagaaacgc	117
rCELSR3	NM_031320.1	gctcttttctgctccttctgc	cagcatggaggtagtggaag	96
rEMR1	NM_001007557.1	ccttgcctgcttcttctggatg	agcatcttgatgttgcgagagc	105
rEMR4	NM_001007558.1	tgtgggactcagtctttctctc	tggagtgtcgtgctggtattc	93
rETL	NM_022294.1	ttgggctcatctataacaaggg	gccgagaatccaacaactacag	88
rGPR110	NA	catacatagggctgggcgtctc	ttgcgtgtgtaggaggtttggc	106
rGPR111	NA	ccctgcccaagtcgtgtctg	agcaagtgtgatgacagcgatg	83
rGPR112	NA	gccttaccaccagagtctttgc	gcactgaatggactgtgaatgg	127
rGPR113	NA	gcgggaggggaaatgcttgc	gcacagcaatgacaaggaccag	110
rGPR114	NA	ccacagtgcccaggtcagtc	cgacgcccatccaggtaagg	84
rGPR115	NA	tcatcggctccaacttcagtgc	agcagggctttgaagagcatcc	121
rGPR116	NM_139110.1	ccaccaaagatgtcactgttcac	gcacagaccactggcttcc	80
rGPR123	NA	ggcagtgttggtggtcatg	ccgttggcatcaagtgtgg	85
rGPR124	NA	ggaggtaggagtgtcaggag	cagtggtacagcggaatcg	104
rGPR125	NA	agggaggacgtgaggctttc	aggtgggaggctggacattg	88
rGPR126	NA	attctgctggattcaggatc	ctgtaccatgaccacgatg	106
rGPR128	NA	accagaaacagacaccattgaaac	tgtggcactgagtccattcc	109
rGPR133	NA	catagggtgtgagcacgactg	cttgatctcgtagggagtcagc	97
rGPR56	NM_152242.1	gcacatgaacctgcttctgg	ggtgcgacaggctgcttc	97
rGPR64	NM_181366.1	gcttctgctcaacctggtcttc	agaaatacagccacggagatgc	87
rGPR97	NA	cctcttcggtgctgtggtgctg	ccctgtcctttgcccgctgtg	81
rLEC1	NM_134408.1	tggatgtgcctagaaggtgtgc	cactgtggcagggaagaggtac	114
rLEC2	NM_022962.1	aacaaggagtcagtagtaatgg	cgcagtgaaagacaaagatg	82
rLEC3	NM_130822.1	cgtgggtcggaatcctgttgtc	aatggtgttacggtcgctctgg	95
rVLGR1	NA	gctgctcttgtgcctctaatg	tcatcgtaacctttccactgtg	92

rHiston protein, 3b*	XM_235304	attcgcaagctccccttcag	tggaagcgcaggtctgttttg	71
rACTIN, beta*	NM_031144	cactgccgcactctcttcct	aaccgctcattgccgatagtg	NA
rTUBULIN, beta*	NM_173102	cggaaggaggcggagagc	agggtgcccatgccagagc	89
rSDCA*	NM_130428	gggagtgccgtggtgtcattg	ttcgcccatagcccccagtag	NA
rCYCLOPHILIN*	NM_008907.1	gagcgttttgggtccaggaat	aatgcccgcaagtcaaagaaa	90
