# Supplementary material for: Adhesion GPCRs are widely expressed throughout the subsections of the gastrointestinal tract
Source: BMC Gastroenterol. 2012 Sep 25;12:134. doi: 10.1186/1471-230X-12-134 (PMC3526421; doi:10.1186/1471-230X-12-134)
Supplement: Additional file 2 — Summary of relative expression of all Adhesion GPCR members. Expression levels relative for each gene (maximal level of expression set to 1; n.d., not detected after 40 cycles of the PCR). The phylogenetic grouping is based on the 7TM regions [3]. GPR111, GPR113, GPR115 were only expressed in one sub-segment: the expression value was arbitrarily set to 1 to indicate the presence of expression. GPR133 and CELSR3 could not be detected in any segment. [file 1471-230X-12-134-S2.pdf]

| Member | Phylogenetic group | E | F | A | D1 | D2 | J1 | J2 | I1 | I2 | C | K1 | K2 |
|---|---|---|---|---|---|---|---|---|---|---|---|---|---|
| BAI1 | I | n.d. | n.d. | n.d. | n.d. | n.d. | n.d. | n.d. | n.d. | 0,48 | n.d. | 0,78 | 1,00 |
| BAI2 | I | n.d. | n.d. | 1,00 | 0,66 | n.d. | n.d. | 0,41 | 0,42 | 0,60 | 0,77 | 0,82 | 0,87 |
| BAI3 | I | n.d. | 0,81 | 0,53 | 1,00 | n.d. | n.d. | 0,22 | 0,26 | 0,25 | 0,15 | 0,32 | 0,32 |
| GPR112 | II | n.d. | n.d. | n.d. | 0,72 | 0,45 | 1,00 | 0,57 | 0,46 | 0,14 | n.d. | 0,11 | n.d. |
| GPR114 | II | n.d. | n.d. | 0,31 | 0,77 | 0,58 | 0,74 | 0,96 | 1,00 | 0,60 | 0,95 | 0,68 | 0,69 |
| GPR126 | II | n.d. | n.d. | n.d. | n.d. | n.d. | n.d. | n.d. | 0,42 | 0,75 | n.d. | 0,72 | 1,00 |
| GPR128 | II | n.d. | n.d. | n.d. | 0,77 | 0,45 | 0,74 | 0,74 | 1,00 | 0,59 | 0,38 | 0,62 | 0,51 |
| GPR64 | II | n.d. | 1,00 | 0,78 | n.d. | n.d. | n.d. | n.d. | n.d. | 0,09 | n.d. | 0,07 | 0,13 |
| GPR56 | II | n.d. | 1,00 | 0,79 | 0,32 | 0,20 | 0,25 | 0,25 | 0,30 | 0,33 | 0,18 | 0,41 | 0,46 |
| GPR97 | II | n.d. | 0,23 | 0,23 | 0,70 | 0,55 | 0,65 | 0,89 | 1,00 | 0,74 | 0,52 | 0,66 | 0,53 |
| VLGR1 | II | n.d. | n.d. | n.d. | n.d. | n.d. | 0,68 | 0,89 | 1,00 | n.d. | n.d. | n.d. | n.d. |
| LEC1 | III | 1,00 | 0,47 | 0,57 | 0,73 | 0,35 | 0,84 | 0,82 | 1,00 | 0,64 | 0,26 | 0,61 | 0,86 |
| LEC2 | III | 0,50 | 0,99 | 0,91 | 0,43 | 0,36 | 0,48 | 0,35 | 0,62 | 0,89 | 0,63 | 1,00 | 0,92 |
| LEC3 | III | 0,71 | 0,74 | 0,53 | 0,80 | 0,61 | 0,60 | 0,84 | 0,81 | 0,77 | 0,86 | 1,00 | 0,56 |
| ETL | III | 1,00 | 0,72 | 0,42 | 0,14 | 0,11 | 0,24 | 0,29 | 0,35 | 0,38 | 0,37 | 0,43 | 0,44 |
| EMR1 | III | 0,55 | 0,44 | 0,53 | 0,47 | 0,51 | 0,62 | 0,92 | 0,83 | 0,71 | 0,99 | 1,00 | 0,91 |
| EMR4 | III | n.d. | n.d. | n.d. | n.d. | n.d. | 0,66 | 0,89 | 1,00 | 0,61 | n.d. | 0,65 | 0,50 |
| CD97 | III | 0,77 | 0,44 | 0,58 | 0,41 | 0,29 | 0,39 | 0,43 | 0,47 | 0,82 | 0,41 | 1,00 | 0,98 |
| GPR123 | IV | n.d. | n.d. | n.d. | n.d. | n.d. | n.d. | n.d. | n.d. | n.d. | n.d. | n.d. | n.d. |
| GPR124 | IV | 1,00 | 0,34 | 0,42 | 0,36 | 0,27 | 0,22 | 0,23 | 0,31 | 0,39 | 0,43 | 0,44 | 0,83 |
| GPR125 | IV | 0,40 | 1,00 | 0,83 | 0,28 | 0,21 | 0,36 | 0,46 | 0,45 | 0,40 | 0,37 | 0,47 | 0,82 |
| CELSR1 | V | 1,00 | 0,62 | 0,58 | n.d. | n.d. | 0,09 | n.d. | n.d. | n.d. | n.d. | 0,12 | 0,51 |
| CELSR2 | V | n.d. | 1,00 | 0,80 | n.d. | n.d. | 0,13 | n.d. | n.d. | n.d. | n.d. | 0,24 | 0,69 |
| CELSR3 | V | n.d. | n.d. | n.d. | n.d. | n.d. | n.d. | n.d. | n.d. | n.d. | n.d. | n.d. | n.d. |
| GPR133 | VI | 0,30 | 0,18 | 0,18 | 0,31 | 0,32 | 0,62 | 0,80 | 1,00 | 0,31 | 0,12 | 0,27 | 0,19 |
| GPR110 | VII | n.d. | n.d. | n.d. | n.d. | n.d. | n.d. | n.d. | 0,85 | 0,92 | n.d. | 1,00 | 0,50 |
| GPR111 | VII | 1,00 | n.d. | n.d. | n.d. | n.d. | n.d. | n.d. | n.d. | n.d. | n.d. | n.d. | n.d. |
| GPR113 | VII | n.d. | n.d. | n.d. | n.d. | n.d. | n.d. | n.d. | n.d. | n.d. | n.d. | 1,00 | n.d. |
| GPR115 | VII | 1,00 | n.d. | n.d. | n.d. | n.d. | n.d. | n.d. | n.d. | n.d. | n.d. | n.d. | n.d. |
| GPR116 | VII | 1,00 | 0,81 | 0,42 | 0,29 | 0,22 | 0,30 | 0,30 | 0,33 | 0,29 | 0,26 | 0,41 | 0,32 |
